# Evolution for Everyone: How to Increase Acceptance of, Interest in, and Knowledge about Evolution

**DOI:** 10.1371/journal.pbio.0030364

**Published:** 2005-12-13

**Authors:** David Sloan Wilson

## Abstract

A success story about teaching evolution: when presented as unthreatening, explanatory, and useful, evolution can be easily appreciated by most people, regardless of their religious and political beliefs or prior knowledge of evolution.

Evolution is famously controversial, despite being as well established as any scientific theory. Most people are familiar with the dismal statistics, showing how a large fraction of Americans at all educational levels do not accept the theory of evolution [1], how efforts to teach evolution often fail to have an impact [2], and how constant vigilance is required to keep evolution in the public school curriculum [3]. Even worse, most people who do accept the theory of evolution don't relate it to matters of importance in their own lives. There appear to be two walls of resistance, one denying the theory altogether and the other denying its relevance to human affairs.

This essay reports a success story, showing how both walls of resistance can be surmounted by a single college course, and even more, by a university-wide program. It is based on a campus-wide evolutionary studies program called EvoS (http://bingweb.binghamton.edu/~evos/), initiated at Binghamton University in 2002, which currently includes over 50 faculty members representing 15 departments. Enthusiasm at all levels, from freshmen students to senior administrators, makes EvoS a potential model for evolution education that can be duplicated; the basic ingredients are present at most other institutions, from small colleges to major universities.

In this essay, I will briefly describe the basic ingredients at both the single-course and program levels. First, however, it is important to document the claim that evolution can be made acceptable, interesting, and powerfully relevant to just about anyone in the space of a single semester.

## Demonstrating Success

The single course is titled “Evolution for Everyone” and does not require any prerequisites. The students who enrolled in fall 2003 came from majors as diverse as anthropology, art, biology, business, chemistry, cinema, computer science, creative writing, economics, education, engineering, english, history, human development, linguistics, management, mathematics, nursing, philosophy, physics, political science, and psychology. The 2003 course was assessed with the help of two experts on evolution education: Dr. Brian Alters, Director of the Evolution and Education Research Center at McGill University (Montreal, Canada), and Dr. Craig E. Nelson, Professor of Biology at Indiana University (Bloomington, Indiana, United States) [4–7]. Information gathered on each student at both the beginning and end of the course included religious and political orientation, prior exposure to evolution education, and an assessment of general thinking skills without reference to specific subject matter. In addition, students wrote short essays throughout the course that were submitted electronically and analyzed for words associated with cognitive operations using the software *Linguistic Inquiry and Word Count* [8,9]. Finally, students assessed the course anonymously in addition to providing information associated with their identity. The details of the assessment are available from the author upon request, and the major results are summarized here.

### Acceptance of, interest in, and knowledge about evolution

**Figure 1:**
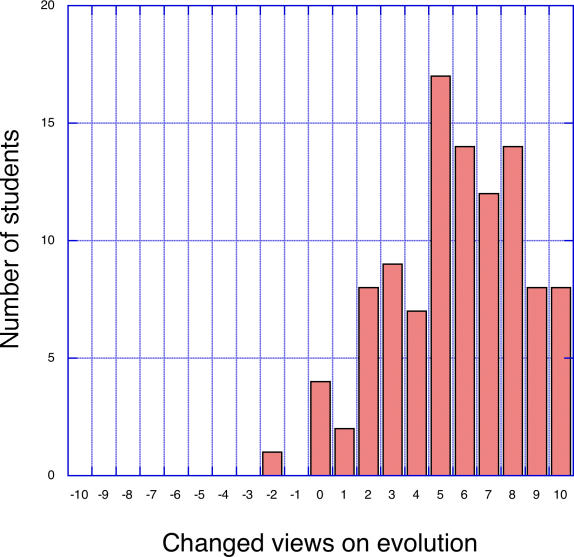
Changed Views on Evolution Anonymous response to the question “How much has this class changed your views on evolution and its relevance to human behavior, on a scale from −10 (negative change) to +10 (positive change)?”

Figure 1 shows the distribution of anonymous responses to the question, “How much has this class changed your views on evolution and its relevance to human behavior, on a scale from −10 (negative change) to +10 (positive change)?” There was a large shift in the positive direction, so much that almost no one who took the course remained unmoved or shifted in the negative direction. The anonymous verbal evaluations speak more eloquently than the numbers: “This course provides evidence that evolution is evident in everything. It revolutionized my way of viewing problems.” “I have always agreed with evolution but I did not know how much of everyday life was affected by it.” “I came into the class not knowing a lot about evolution. I now have an entirely new outlook on how evolution can be applied to many aspects of life.” The positive anonymous evaluations are also reflected in the before-and-after measurements gathered on each student, and become even more interesting when related to the background variables.

### Political orientation

Evolution has often been used to support conservative political ideologies, to the dismay of liberal thinkers. It might seem that politically conservative students would embrace the course material more enthusiastically than the liberals, but this was not the case. The course was equally effective across the political spectrum.

### Religious orientation

The average student was moderately religious, and variation spanned the range from committed atheists to committed believers. Numerous students wrote at length about their religious upbringing and values in their first assigned essay on the topic, “What I know about evolution and its relevance to human affairs.” Surely, the famous tension between evolution and religion should be reflected in the course assessment measures. Remarkably, it was not. The course was effective across the spectrum of religious belief.

### Prior evolution and science education

The average student had at least some exposure to evolution in high school, and variation spanned the range from no exposure to prior college courses. There was also extreme variation in exposure to science education, as had been expected from the diversity of majors. Remarkably, the course was again effective across the entire spectrum. What the students gained from the class did not require, and was not provided by, prior science and evolution education.

### General cognitive development

As outlined in more detail below, the course involved first teaching a set of basic principles and then applying them to a broad range of topics. This experience increased general thinking skills as well as specific knowledge about evolution, according to before-and-after measurement of critical thinking and increase in the frequency of words indicative of cognitive operations in the essays over the course of the semester. Anonymous verbal evaluations such as “this course…has revolutionized my way of viewing problems” clearly reflect more than a body of facts learned about a particular subject.

The assessment did not include a comparison with another course, because it is difficult to know what an appropriate control group would be. The most relevant comparisons are provided by the internal analysis, especially the before-and-after comparisons for single individuals and comparison of individuals who differ in their background variables. Undoubtedly, there are students who didn't take the course that would have been less receptive to the material, including some committed to creationism, but the course clearly comes close to living up to its name, “Evolution for Everyone.” Now that the success of the course has been documented, we can examine the ingredients that make it work.

## How It Works

Alters and Nelson have written on the need for science education to go beyond strict lecture mode and to teach the scientific process in addition to factual material [5–7]. “Evolution for Everyone” employs as many of these techniques as possible, some of which will be described below. However, the main ingredients of success involve teaching a sequence of ideas.

### Beginning with implications

The main problem with accepting evolution involves implications, not facts. Threatening ideas are like other threats—the first impulse is to run away or attack them. Make the same ideas alluring, and our first impulse is to embrace them and make them our own. Neither impulse is very respectable scientifically. After all, scientists are supposed to accept ideas when they are true, regardless of their consequences. Nevertheless, the key to making evolution a subject that anyone can understand and everyone should want to understand is to focus first on the implications. A good theory should do two things. First, it should explain the world as it has existed in the past and exists in the present. Second, it should provide ways to improve the world in the future. The first major idea to convey is that evolution is a good theory by both of these standards.

This requires a discussion of past threatening associations, even before the theory is presented. Evolution has been associated with immorality, determinism, and social policies ranging from eugenics to genocide. It has been used to justify racism and sexism. All of these negative associations must be first acknowledged and then challenged. It's not as if the world was a nice place before Darwin and then became mean on the basis of his theory. Before Darwin, religious and other justifications were used to commit the same acts, as when the American colonists used the principle of divine right to dispossess Native Americans, and men claimed that women were designed by “God and Nature” for domestic servitude. These beliefs are patently self-serving and it should surprise no one that an authoritative scientific theory would be pressed into the same kind of service. It is the job of intellectuals to see through such arguments and not be taken in by them. Moreover, the deep philosophical issues associated with topics such as morality, determinism, and social equality are increasingly being approached from a modern evolutionary perspective and are among the topics to be discussed in the course. When these issues are discussed at the beginning of the course, students put their own threatening associations with evolution on hold and become curious to know how a subject that they associate with science (evolution) can shed light on a subject that they associate with the humanities (philosophy). Students who indicate exceptional interest are referred to books that are both authoritative and accessible, such as Daniel Dennett's *Darwin's Dangerous Idea* [10–15].

### Adaptationism—A third way of thinking

The next task is to formally present the concept of natural selection. The principles of phenotypic variation, corresponding variation in fitness, and heritability are so simple and seemingly inevitable in their consequences that the main question is not “What are they?” or “Are they true?” but “Why should they be regarded as such a big deal?” To answer this question, I ask the students to imagine how someone would explain the properties of an organism before Darwin's theory of evolution. Only two options would be available; theological (God's handiwork) or material (explaining the properties of the whole from the properties of the component parts). The big deal about natural selection is that it provides a third explanatory framework, different from both theology (this is already obvious to the students) and materialism (this is not). To the extent that the material composition of organisms results in heritable variation, it becomes a kind of living clay that can be molded by environmental forces that influence survival and reproduction. The most interesting properties of a clay sculpture are caused by the molding action of the artist, not the physical properties of clay. In the same way, evolutionary biologists routinely make predictions about the properties of organisms (such as “many prey organisms match their background to avoid detection by predators”) without any reference to the physical materials of the organisms, including their genes.

This is the fundamental distinction between proximate and ultimate causation in evolutionary biology, and it is the second major idea in the sequence that I attempt to convey to students. The distinction is important because it has such predictive value. Knowing only a little about an organism and its environment, one can make predictions about its properties that are not certain to be correct, but which are likely to be correct. In mundane terms, they are good guesses. I make this point with a class exercise of the sort recommended by Alters and Nelson. Choosing the subject of infanticide, I say that superficially it might seem that organisms would never evolve to kill their own offspring, but with a little thought the students might be able to identify situations in which infanticide is biologically adaptive for the parents. I ask them to form small groups by turning to their neighbors to discuss the subject for five minutes and to list their predictions on a piece of paper.

After the lists are collected, I ask the students for some of their predictions to list in front of the whole class. They are eager to talk, and reliably identify the three major adaptive contexts of infanticide: lack of resources, poor offspring quality, and uncertain paternity, along with less likely possibilities, such as population regulation, that can be set aside for future discussion. I conclude by attempting to convey the simple but profound message of the exercise: How can they, mere undergraduate students, who know almost nothing about evolution and (one hopes) know nothing at all about infanticide, so easily deduce the major hypotheses that are in fact employed in the study of infanticide for organisms as diverse as plants, insects, and mammals? That is just one example of the power of thinking on the basis of adaptation and natural selection.

### One explanatory framework, many applications

The next major idea to convey is that the same reasoning can be applied to an infinite number of topics. Why are males larger than females in some species and the reverse in others? Why are there two sexes in the first place? Why are males and females born in equal proportions in some species but not others? Why do some organisms reproduce once and then die, while others reproduce at repeated intervals? Why do some plants live for three weeks and others for 3,000 years? Why are some organisms social and others solitary? Among social organisms, why do some individuals cooperate and others exploit? Predictions based on natural selection provide a starting point for inquiry on all of these subjects, just as with infanticide. Evolutionary theory provides an escape from the extreme specialization that characterizes so much of the rest of science. It transcends taxonomic boundaries because organisms as different as plants, insects, and mammals can be similar in terms of their adaptations to similar environmental problems, for infanticide and many other subjects. It transcends subject boundaries because the problem of how to select food (for example) is very similar to the problem of how to select a mate. Evolutionary biologists sometimes take it for granted that they possess a common language that can be spoken across so many domains of knowledge. It is an extraordinary fact and needs to be presented as such to students learning about evolution for the first time.

### Humans in addition to the rest of life

One of the biggest tactical errors in teaching evolution is to avoid discussing humans or to restrict discussion to remote topics such as human origins. The question of how we arose from the apes is fascinating and important, but is only one of any number of questions that can be asked about humans from an evolutionary perspective—including infanticide. If evolutionary theory can make sense of this subject for organisms as diverse as plants, insects, and mammals, what about us? If we operate by different rules than all other creatures for this and other subjects, why should this be so? The most common answer to this question is “learning and culture,” but what exactly are these things? Do they exist apart from evolution, or do they themselves need to be explained from an evolutionary perspective? I raise these issues early in the course, not to answer them, but to emphasize how much is “on the table” as part of the course.

For millennia, humans have regarded themselves as categorically different from other creatures in their mental, moral, and aesthetic abilities. We are obviously unique in some respects, but in exactly what way needs to be completely rethought. Nonhuman species have been discovered to be vastly more sophisticated and behaviorally flexible than most people imagined even 30 years ago. They solve the recurrent problems of their environments as well as, or better than, humans. They can change not only their behaviors but their entire bodies and life histories in response to environmental change. Something happened several million years ago to give our species a special kind of behavioral flexibility, and the ability to socially transmit behaviors in a cumulative fashion (culture). A sophisticated knowledge of evolution is required to discover exactly what happened. As for the consequences of these new mental capacities, they do not necessarily cause our species to play by a different set of rules than other species. Perhaps they enable us to play the evolutionary game better and faster than other species. For a specific topic such as infanticide, it all boils down to an empirical question: Do people commit infanticide under the same environmental conditions as other species? It turns out that there is a sizeable literature for this subject, to be reviewed later in the course along with a more general discussion of the nature of human learning and culture. Students who become exceptionally interested are directed to a growing genre of accessible and authoritative books, such as Jared Diamond's *Guns, Germs, and Steel* [16–22].

It might seem that boldly discussing subjects such as human infanticide (which the students quickly connect to the contemporary issue of abortion), along with other topics such as sex differences and homosexuality later in the course, is the ultimate in political incorrectness. However, I have taught this material for many years in prior courses without a single complaint, and the assessment of “Evolution for Everyone” demonstrates an overwhelmingly positive response across the religious and political spectrum. Clearly, there is a way to proceed that arouses intense interest without animosity or moral outrage. In the case of infanticide, evolutionary theory doesn't say that it's right—it is used to make an informed guess about when it occurs. All of the students want to know if the guess proves to be correct for humans in addition to other creatures, regardless of their moral stance on abortion. Moreover, they see that the information can be useful for addressing the problem, whatever particular solution they have in mind. The importance of culture is not denied, but becomes part of the evolutionary framework rather than a vaguely articulated alternative. The picture that emerges makes sense of cases of infanticide that appear periodically in the news (typically young women with few resources and under the influence of a male partner who is not the father) and that previously seemed inexplicable. Nearly everyone values this kind of understanding and thinks that it can be put to positive use, as demonstrated by the quantitative assessment. More generally, including humans along with the rest of life vastly increases students' interest in evolution and acceptance to the degree that it seems to lead to understanding and improvement of the human condition.

### Not everything is adaptive

Readers of this essay familiar with evolutionary theory might be wondering why my sequence of ideas relies so heavily upon adaptation and natural selection up to this point. Isn't there more to evolution than natural selection, as Stephen Jay Gould cautioned at every opportunity [23–25]? The answer is “yes,” but this point needs to come later in the sequence, after the basic concept of adaptation and its explanatory power have been established. There are many reasons why organisms are not perfectly adapted to their environment. There might be insufficient time, especially when the environment changes, as it does with a vengeance in our own species. The living clay of heritable variation is by no means infinitely malleable. There are hidden connections among traits based on genetics and development, such that selection for one trait drags others along. Gene frequencies change by drift and mutation in addition to selection. The list goes on and on, and mature research programs in evolutionary biology pay attention to all of these factors.

If so, then why should adaptation and natural selection enjoy a special status? The answer is quite practical: It is usually much easier to make a prediction based on knowledge of the organism in relation to its environment than predictions based on the other factors. In the case of infanticide, my students easily derived the major adaptationist predictions, but would be at loss to derive predictions based on phylogeny, developmental and genetic constraints, neural mechanisms, and so on. This asymmetry in the ease of making predictions, combined with the admitted importance of the hard-to-predict factors, leads to proper understanding of the adaptationist program [26]. It is not a claim that everything is adaptive, but an effective method of scientific inquiry that begins with an adaptationist hypothesis as the best first guess, with the full expectation that it will be partially wrong due to the many hard-to-predict factors. Partial failures are then used as guide for the identification of other factors. This is not the only way to conduct evolutionary science, but I have used it as an effective way to order the sequence of ideas pedagogically. The Gouldian paradigm does not come first, but it does occupy center stage for a section of the course with the intention of making it a permanent part of the conceptual framework being built.

### Evolutionary adaptations are not always benign

Even when organisms are highly adapted to their environments, their properties do not always correspond to the intuitive notion of adaptation. Everyone can agree about the impressive design of a butterfly that exactly resembles a leaf, or a fish shaped to cruise effortlessly though the water, but how about a species that degrades its own habitat or a social partner who fails to cooperate? Fitness is a relative and local concept. It doesn't matter how well an organism survives and reproduces, only that it does so better than other organisms in its vicinity. As a result, many evolutionary adaptations appear selfish and shortsighted in human terms, creating problems at larger temporal and spatial scales.

If behaviors regarded as immoral in human terms are adaptive and “natural,” then aren't all the fears about evolution justified? No—because behaviors that are regarded as moral in human terms are also adaptive and “natural” under the right circumstances, which can be illustrated with the following exercise of the sort suggested by Nelson and Alters. First, the class is asked to list the behaviors that they associate with morality. The most common items include altruism, honesty, love, charity, sacrifice, loyalty, bravery, and so on. Then they are asked to list behaviors that they associate with immorality, and respond with opposite items such as selfishness, deceit, hatred, miserliness, and cowardice. With these lists in mind, the students are asked three questions: (1) What would happen if you put a single moral individual and a single immoral individual together on a desert island? (The students quickly conclude that the moral individual would become shark food within days.) (2) What would happen if you put a group of moral individuals on one island and a group of immoral individuals on another island? (The students are equally quick to conclude that the moral group would work together to escape the island or turn it into a little utopia, while the immoral group would self-destruct.) (3) What would happen if you allow one immoral individual to paddle over to Virtue Island? (The answer to this question is complex because it is a messy combination of the straightforward answers to the first two questions.)

This exercise is simple and entertaining, but profound in its implications. It shows that most of the traits associated with human morality can be biologically adaptive. Groups of moral individuals are likely to survive and reproduce better than any other kind of group. The problem with morality is its vulnerability to subversion from within. To the extent that natural selection is based on fitness differences *within* groups, behaviors associated with immorality are the expected outcome. To the extent that natural selection is based on fitness differences *among* groups, behaviors associated with morality are the expected outcome (these statements apply to all evolutionary models of cooperation and altruism when the relevant groups are appropriately defined, including inclusive fitness theory, evolutionary game theory, and multilevel selection theory) [27,28]. The discerning student quickly perceives a disturbing corollary: Can't behaviors that count as moral within groups be used for immoral purposes among groups? The answer to this question is “yes,” which means that moral conduct among groups is a different and more difficult evolutionary problem to solve than moral conduct within groups [14,27].

The important point is that evolutionary theory can potentially explain the evolution of behaviors associated with morality and immorality. This is vastly different than the usual portrayal of evolution as a theory that explains immorality but leaves morality unaccounted for. The average student is well aware that immoral behaviors usually benefit the actor, that human groups have a disturbing tendency to confine moral conduct to their own members, and so on. When evolutionary theory is presented as a framework for understanding these patterns in all their complexity, including the good, the bad, the beautiful, and the ugly, it is perceived as a tool for understanding that can be used for positive ends, rather than as a threat. These issues are discussed in more detail later in the course. In the initial sequence of ideas, it is important to establish that evolutionary adaptations are not always adaptive in the everyday sense of the word, and that societal adaptations in particular require special conditions to evolve.

### Using the framework

At this point (about mid-semester), the students are told that they have acquired a conceptual framework that can be used to study virtually any subject in biology and human affairs, which will be used to study particular topics for the rest of the semester. There is great flexibility in the topics that can be chosen, which is facilitated by having the students read, rather than a textbook, well-chosen articles from the primary scientific literature. I begin with the subject of Darwinian medicine; it is intrinsically interesting, illustrates a number of general principles, and is directly relevant to students preparing for careers in the health sciences.

The health sciences are enormously sophisticated in the study of proximate mechanisms but often ignorant of evolutionary principles, as pointed out by G.C. Williams and R. Nesse in their influential scientific article, “The Dawn of Darwinian Medicine” [29] and popular book *Why We Get Sick* [30]. Simply put, most doctors and medical researchers don't know what the students have learned during the first half of the course. I begin by assigning two articles from the primary scientific literature, one on pregnancy sickness [31] and the other on the anti-microbial properties of spices [32]. Both topics strike the students as arbitrary, as if they were pulled out of a hat. Yet each turns out to be a fascinating scientific detective story enlightened by the evolutionary principles that they learned during the first half of the course. The tendency of women to become nauseated during the first trimester of pregnancy has been treated by doctors as a sickness to be cured with medicine. In fact, it is an important biological adaptation that causes the mother to avoid foods that would damage her developing fetus. The use of spices to flavor food seems like an aspect of culture without any biological basis. In fact, most spices have important antimicrobial properties and their use within and among cultures is proportional to the likelihood of food spoilage. Both articles are authored by Paul Sherman, an evolutionary biologist without any prior training in either specific subject. Remarkably, both of his co-authors were undergraduate students when the papers were researched and written. How could they make such important contributions to knowledge without years of specialized training? They used evolutionary theory to ask the right questions, just as my students were able to do for the subject of infanticide.

After these two articles on specific topics, I assign the more general article on Darwinian Medicine by Williams and Nesse [29]. I also have the students make their own search for scientific articles using the key words “Darwinian medicine,” and ask them to post abstracts on the course Web site. This section of the course reveals the existence of a new scientific field that the students can understand and to which they can potentially contribute, even as undergraduates.

The section on Darwinian medicine is followed by sections on other topics, including violence, sexuality, personality, and culture. Like medicine, these subjects are voluminous and sophisticated in their own ways but are often ignorant of basic evolutionary principles, enabling foundational insights to be made that the students can easily appreciate. They realize that they have started to approach the study of humans in the way that evolutionary biologists approach the rest of life, with a common language that can be spoken across many domains of knowledge.

### A subject of their own

The final vital ingredient of the course is to have the students choose their own topic to explore from an evolutionary perspective. This can be done in several ways depending upon class size and available resources. In my case, I form the students into small groups supervised by undergraduate teaching assistants, culminating in a poster session that emulates a scientific conference at the end of the semester. Most of the students become highly motivated to study “their” topic from an evolutionary perspective; in 2003, topics included adoption, alcoholism, attractiveness, body piercing, depression, eating disorders, fashion, fear, hand dominance, homosexuality, marriage, play, sexual jealousy, sibling rivalry, social roles, suicide, video games, and yawning. The topics were posted on the course Web site and students visited each other's posters during the session, providing yet another demonstration of how evolutionary theory can be used to approach a diversity of subjects.

To summarize, “Evolution for Everyone” works by establishing a general conceptual framework through a sequence of ideas. The framework is then strengthened and consolidated by applying it to a number of specific topics. Virtually all students respond to the class because they cease to be threatened by evolutionary theory and begin to perceive it as a powerful way to understand and improve the world. Once the theory becomes alluring, the only remaining obstacle to learning is the intrinsic difficulty of the subject. That, it turns out, is not much of an obstacle either. Almost anyone can master the basic principles of evolution and incorporate them into their own thinking, providing both a foundation and an incentive to advance their knowledge in subsequent courses.

## From a Single Course to a Campus-Wide Program

Students who “catch the evolution bug” are usually eager to pursue their newfound interests. At Binghamton University we were able to assist them by creating a campus-wide program that can be replicated at other institutions—in spirit if not in each and every detail. The best way to learn about EvoS is by visiting its Web site (http://bingweb.binghamton.edu/~evos/), but the most basic ingredients can be summarized as follows.

### An initial faculty core

Most colleges and universities have at least some faculty who are already teaching and conducting research from an evolutionary perspective. At Binghamton, we had core groups in the biology, anthropology, and psychology departments, and single individuals in other departments such as economics and philosophy. An initial faculty core can get the program going and can benefit personally by enhancing interactions with each other.

### Organize existing resources

Core faculty already teach permanent courses from an evolutionary perspective, although students in a given department are usually unaware of offerings in other departments. In addition to their permanent courses, most active faculty teach special topic seminars on new subjects that interest them, involve students in their research, and so on. Most departments and higher administrative units provide modest funds for seminar speakers and new educational initiatives. These existing resources can be organized so that the whole is much more than the sum of the parts.

### Maximize accessibility and minimize additional workload

At Binghamton it is possible for both undergraduate and graduate students to take a course of study that results in a certificate that accompanies their degree. Unlike a second major or a minor, both of which impose severe additional course loads on the student, a certificate program allows a given course to simultaneously count toward the certificate and one's major or graduate degree. This is important because many EvoS students are already “turned on” in other respects, with a minor or double major that would prevent them from adding still more. A certificate program is relatively easy to implement administratively (at least at Binghamton), can be integrated with existing course requirements, and is accessible to all students.

### Depth and breadth

EvoS is designed both to increase competence in one's chosen subject area (depth) and to transcend disciplinary boundaries (breadth). Breadth is achieved in part through the EvoS seminar series that will be described in more detail below. Depth is achieved by having an EvoS faculty advisor help each student develop a curriculum tailored to his or her interests from the menu of offerings.

### Growing the program

Expanding the program beyond its initial core requires confronting some uncomfortable truths about the status of evolutionary theory in academia. Earlier, I said that there are two walls of resistance to evolution, one that denies its validity altogether and another that denies its relevance to human affairs. It is easy for academics to ridicule the first wall (creationism and its born-again cousin, intelligent design), but the second wall has existed within academia for most of the 20th century, shaping the history of all human-related subjects. The wall is still staunchly maintained in some quarters, but even the most open-minded scientists and scholars are handicapped by the barriers separating evolutionary theory from their disciplines in the past. The most important developments in human-related research from an evolutionary perspective have taken place within the last 20 years, and weren't even on the radar when many faculty members were receiving their own graduate training. Expanding the program therefore requires faculty training in addition to student training.

Fortunately, it is possible to do this without imposing an unacceptable additional workload on the faculty. EvoS faculty participants are not all experts on evolution. Even better, they include some experts and others who have adopted the same receptive attitude as the students, resulting in the accumulation of expertise as the program develops. EvoS has already stimulated teaching and research activities in new subject areas, involving faculty members who were not part of the initial core. When the evolutionary perspective proves its worth to a faculty member, achieving a professional level of competence becomes a priority that contributes to rather than detracting from their career goals.

### The university as a single intellectual community

The spirit of our campus-wide program is perhaps best represented by the EvoS seminar series, which brings an external speaker to campus at approximately two-week intervals. Table 1 lists a sample of speakers from the 2004–2005 academic year, which illustrates three points. First, the speakers span the length and breadth of the biological sciences, the human behavioral and social sciences, and the humanities. Second, the speakers include some of the most distinguished members of their respective fields in addition to up-and-coming young scientists. Third, the seminars are not “watered down” for a general audience, but are much the same as the speakers would give in departmental seminars at other universities. Nevertheless, all of the seminars are attended, understood, and enjoyed by a single audience of undergraduate students, graduate students, and faculty representing all departments on campus. The only thing that makes this possible is theoretical integration. The speakers and audience alike share a common conceptual framework that enables them to transcend disciplinary boundaries.

**Table 1:**
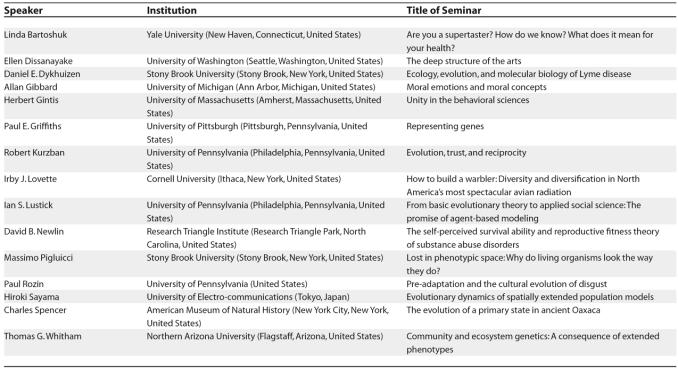
A Sample of Speakers in the Campus-Wide EvoS Seminar Series during the 2004–2005 Academic Year

The EvoS seminar series simultaneously performs a number of important functions. It is advertised campus-wide, and speakers are usually cohosted with the most relevant department, which means that any given talk is attended by a mix of EvoS participants who attend all the talks and non-participants who are attracted by a particular speaker or topic and who encounter the evolutionary perspective for the first time. It facilitates research collaborations between Binghamton University faculty and graduate students, and outside experts. Finally, it is used as the basis for a two-credit course that must be taken twice to earn the EvoS certificate. Students in the course read one or more papers and post an electronic commentary in preparation for each seminar, attend the seminar, and attend an informal dinner and continuing discussion with the speaker that follows each seminar. The dinner and continuing discussion provide a rich social and intellectual experience that is repeated for a different specific topic with each seminar. Little wonder that many EvoS students regard EvoS as their academic “home” rather than their particular department. As one student put it, “EvoS provides a stimulating atmosphere within which biologists, psychologists, anthropologists, philosophers, social scientists, and even those in the arts can transcend traditional academic boundaries and collaborate in addressing mutually interesting questions. It creates a think-tank atmosphere of sorts, and it's a beautiful thing!”

In many ways, this type of experience approaches the ideal of a liberal arts education. It should be especially appealing to small colleges that have difficulty achieving a critical mass in single subject areas. Evolutionary theory is not the only common language, but it is a very good one that will eventually become part of the normal discourse for all subject areas relevant to human affairs and the natural world. Much can be done to facilitate this process, and EvoS provides one effective model. When it comes to evolution and teaching evolution, the future can be different from the past.
